# Analysis of Electromagnetic Properties of New Graphene Partial Discharge Sensor Electrode Plate Material

**DOI:** 10.3390/s22072550

**Published:** 2022-03-26

**Authors:** Huiyuan Zhang, Zhensheng Wu

**Affiliations:** School of Electrical Engineering, Beijing Jiaotong University, Beijing 100044, China; zhshwu@bjtu.edu.cn

**Keywords:** graphene, partial discharge, electrode, conductivity, dielectric constant, refractive index

## Abstract

Advanced sensing and measurement technology is the key to realizing the transparent power grid and electric internet of things. Meanwhile, sensors, as an indispensable part of the smart grid, can monitor, collect, process, and transmit various types of data information of the power system in real-time. In this way, it is possible to further control the power system. Among them, partial discharge (PD) sensors are of great importance in the fields of online monitoring of insulation condition, intelligent equipment control, and power maintenance of power systems. Therefore, this paper intends to focus on advanced sensing materials and study new materials for the improvement for partial discharge sensors. As two-dimensional material, graphene is introduced. The electromagnetic properties of graphene partial discharge sensor electrode plate material are analyzed theoretically. By studying the influence of different chemical potential, relaxation time, temperature, and frequency, we obtain the changing curve of conductivity, dielectric constant, and refractive index. A linear regression model based on the least-squares method was developed for the three electromagnetic properties. Finally, the simulation and experiment verified that the graphene partial discharge sensor has better absorption of the partial discharge signal. This study can apply to the design of graphene partial discharge sensors.

## 1. Introduction

High-voltage switchgear is an important type of transmission and distribution equipment, and its operational safety directly affects the reliability of the power system [1]. Partial discharge is the main cause of insulation deterioration and insulation failure of switchgear [2]. The safe and reliable operation of switchgear determines the reliability and safety of the power system, and switchgear has a pivotal position in the power system. Therefore, for the safe operation of switchgear, the monitoring of partial discharge is of great importance [3,4].

At present, PD detection methods mainly include the pulse current method (PCM), ultrahigh-frequency (UHF) method, ultra-acoustic wave (UAW) method, optical detection method, and transient Earth voltage (TEV) method [5]. Many famous companies and scientific research institutions have been competing to improve partial discharge monitoring systems. They have made progress in the research of various internal and external antennas, and UHF sensors. Ref. [6] proposes a novel PD detection method for transformers on-site adopting its component as a UHF sensor. In [7], a UHF antenna with a relatively small aperture and radome for PD detection was designed. In [8], a microstrip antenna was designed with a radiating element shape based on the leaf of the *Jatropha mollissima (Pohl) Baill* plant for PD detection. Ref. [9] proposes an innovative online PD detection system and a corresponding application strategy based on intelligent feedback distributed TEV wireless sensor network. The above improvements are limited by the traditional metal sensing materials, and new advanced sensing materials are urgently needed to support them. Therefore, this paper intends to study the new partial discharge sensor based on new advanced sensing material. Since the structure of the TEV partial discharge sensor is relatively simple and easy to dissect, it is proposed to carry out the research on the new advanced sensing material partial discharge sensor starting from the TEV sensor.

With the continuous research and development of new materials, many advanced materials are researched and prepared by researchers and further applied in the field of engineering. In [10], the layer-dependent frictional character is examined for the buried graphene nanopores, which can be used as solid lubricants. In [11], this study can be used to develop high-performance anodes with high-fraction active sodium-based dual-ion batteries (Na-DIBs) materials, which show potential for large-scale energy storage applications. Ref. [12] designed FeCoSiB/Pb(Zr,Ti)O_3_ magnetoelectric composite as a magnetic sensor, which can be used in the field of gear speed measurement. As a typical representative of two-dimensional planar superconducting materials, the special energy band structure of graphene gives it a series of excellent properties such as high conductivity, low magnetic permeability, and high dielectric constant [13]. By microscopic regulation for graphene materials, more sensing materials with excellent electrical properties can be explored. It has a strong competitive ability compared with traditional sensing materials.

In the field of engineering, many experts and scholars have conducted extensive research on the excellent properties of graphene sensor devices. Ref. [14] proposes a novel graphene-based Hall sensor using an alternating current (AC) modulated gate voltage. In [15], dynamic measurements of MR on devices with a low degree of extrinsic disorder lead to stable and reliable single-layer graphene magnetosensors. In [16], a new type of space radiation sensor with small volume, high resolution, and radiation resistance was proposed. However, graphene has not been reported in the field of partial discharge measurements.

Graphene differs from ideal conductors. Its electromagnetic properties change with frequency, which can enhance electromagnetic wave absorption. Ref. [17] proposes a terahertz (THz) graphene metamaterial absorber (MA), which has the advantages of tunability, high absorption, and broadband. In [18], the polyaniline nanorods/graphene sheets composites were synthesized with enhanced microwave absorption properties. In [19], a novel gate tunable graphene-metal hybrid plasmonic antenna with stacking configuration is proposed, which has a broad range of frequencies with enhanced absorption properties. The above studies only focus on high-frequency electromagnetic waves, however, but few studies have considered the absorption of low-frequency electromagnetic waves generated by partial discharge sensors.

Traditional TEV sensors only use the principle of capacitive voltage division to measure transient earth waves. This approach does not take into account the absorption of electromagnetic waves by the sensor itself. To further improve traditional PD sensors, this paper proposes the study of the electromagnetic properties of the electrode plate material of the new graphene partial discharge sensor. The electromagnetic properties, including conductivity, dielectric constant, magnetic permeability, and refractive index, are derived theoretically. We also analyze the influence of electrochemical potential, temperature, frequency, and relaxation time on electromagnetic properties. Based on the least-squares method, the prediction model of electromagnetic properties is established. Finally, by simulation and experiment, the partial discharge signal absorption properties of graphene partial discharge sensor and traditional partial discharge sensor are compared. This will lay the foundation for further research on the design of graphene partial discharge sensors.

## 2. Electromagnetic Properties

When partial discharge occurs in the switchgear, the partial discharge can produce time-varying electromagnetic waves. It is are usually seen as a source of pulse line current at a certain point. When it radiates outward, the frequency of electromagnetic signals can reach GHz. The electromagnetic wave radiates in the form of spherical waves and propagates in various media [20]. Therefore, this process satisfies Maxwell’s differential equations.

Therefore, to analyze the propagation law of electromagnetic waves in PD sensors accurately, Maxwell’s equations must be used. Meanwhile, the analysis of electromagnetic properties is particularly important. As for electromagnetic materials, the description of electromagnetic properties mainly needs to start from electrical conductivity, magnetic permeability, and dielectric constant. Considering the electromagnetic properties of graphene copper-clad electrode plates are particularly complex, the electromagnetic properties of the new graphene PD sensor electrode plates were studied.

### 2.1. Electrical Conductivity

Electrical conductivity is an important parameter for analyzing electromagnetic properties. Japanese scientist Kubo has proposed the relationship between energy level spacing and metal particle diameter and gave the famous Kubo formula. The Kubo equation for the electrical conductivity of graphene is [21]

(1) $$\sigma \left(w,\mu_{c},\Gamma ,T\right)=\frac{ie^{2}\left(w-i2\Gamma \right)}{\pi \hbar^{2}}\left[\frac{1}{{\left(w-i2\Gamma \right)}^{2}}\int_{0}^{\infty }a\left(\frac{\partial f_{d}\left(a\right)}{\partial a}-\frac{\partial f_{d}\left(-a\right)}{\partial a}\right)-\int_{0}^{\infty }\frac{f_{d}\left(-a\right)-f_{d}\left(a\right)}{{\left(w-i2\Gamma \right)}^{2}-4{\left(a/\hbar \right)}^{2}}da\right]$$

### 2.2. Dielectric Constant

For substance, the dielectric constant is a measurement of the ability to increase the capacitance concerning vacuum. The dielectric constant increases with increasing molecular dipole moment and polarizability. The relative dielectric constant is the ratio of dielectric constant *ε* and the vacuum dielectric constant ε_0_.

In this article, the dielectric constant mainly refers to the relative dielectric constant. It is important data to characterize the electrical properties of dielectric or insulating materials. Commonly used vacuum dielectric constant ε_0_ = 8.854187817 × 10^−12^ F/m.

### 2.3. Magnetic Permeability

Magnetic permeability is a physical quantity that characterizes the magnetic properties of a magnetic medium. It represents the ability to hinder magnetic flux after current flowing, or the ability to conduct magnetic lines [21]. The relative permeability of the magnetic medium is usually used, which is defined as the ratio of the magnetic permeability μ and the vacuum permeability μ_0_. Commonly used vacuum magnetic permeability μ_0_ = 4π × 10^−7^ H/m.

### 2.4. Refractive Index

The refractive index is closely related to the electromagnetic properties of the medium. According to classical electromagnetic theory, the refractive index is related to frequency, called the dispersion phenomenon. The refractive index of air is very close to 1 for waves of various frequencies.

## 3. Electromagnetic Properties of Graphene Copper-Clad

**Figure 1:**
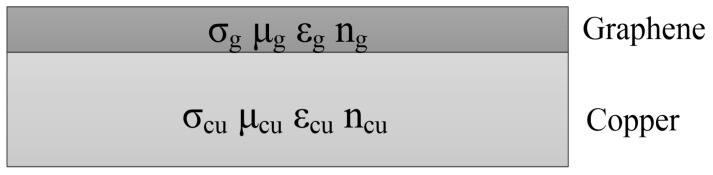
Electrode plate model.

In the study of the electromagnetic properties of graphene copper-clad electrode plate materials, we choose to analyze the electromagnetic properties of graphene and copper separately. We mainly focus on the electromagnetic properties of graphene, due to its more complex characteristics. The electrode plate model of graphene PD sensor is shown in Figure 1.

### 3.1. Electromagnetic Properties of Graphene

Graphene is dispersive material, and its electromagnetic characteristic parameters are corresponding functions with the change of frequency. Meanwhile, due to the special frequency domain expression, the electromagnetic properties of graphene are very complex. The electromagnetic parameters of graphene, such as conductivity, dielectric constant, and refractive index, are mainly related to frequency *w*, chemical potential *μ*_c_, scattering rate Γ, temperature *T*, and other factors. Its conductivity can be calculated by Kubo’s formula, and then the dielectric constant and refractive index can be further calculated by the conductivity.

#### 3.1.1. Electrical Conductivity of Graphene

Because of the hybridization of carbon atoms, the unique gapless electron energy band structure makes graphene exhibit metallic properties.

The two-dimensional characteristics of graphene materials imply their inherent anisotropy in three-dimensional space. Assuming that the graphene sheet is perpendicular to the *z*-axis, the anisotropy of graphene can be modeled by the following equation:

(2) $$\overset{↔}{\sigma }=\left[\begin{array}{ccc} \sigma_{xx} & \sigma_{xy} & 0\\ \sigma_{yx} & \sigma_{yy} & 0\\ 0 & 0 & 0 \end{array}\right]$$

In the most practical applications, graphene exhibits many symmetries. Therefore, the above equation can be simplified by the following equation:

(3) $$\begin{array}{l} \sigma_{xy}=\sigma_{yx}=0\\ \sigma_{xx}=\sigma_{yy}=0 \end{array}$$

In this case, the complex surface conductivity of graphene can be derived from the Kubo equation. The electrical conductivity of graphene consists of intra-band conductivity *σ*_intra_ and inter-band conductivity *σ*_inter_, both of which are very important electromagnetic parameters. By the Kubo formula, it can be obtained as [22]:

(4) $$\sigma_{g}=\sigma_{\mathrm{intra}}+\sigma_{\mathrm{inter}}$$

When the graphene layers number *N*_g_ is small, the total conductivity can be written as

(5) $$\sigma_{g}=N_{g}\left(\sigma_{\mathrm{intra}}+\sigma_{\mathrm{inter}}\right)$$

where the intra-band conductivity *σ*_intra_ represents the scattering process of electrons and photons, which can be expressed as

(6) $$\sigma_{\mathrm{intra}}=\frac{ie^{2}}{\pi \hbar^{2}\left(w-i2\Gamma \right)}\int_{0}^{\infty }a\left(\frac{\partial f_{d}\left(a\right)}{\partial a}-\frac{\partial f_{d}\left(-a\right)}{\partial a}\right)da=\frac{ie^{2}}{\pi \hbar^{2}\left(w-i2\Gamma \right)}F_{1}$$

where *e* represents the charge and *ħ* = *h*/2π represents the angular Planck constant. k_B_ is the Boltzmann constant, *a* is the energy, and *F*_1_ represents the simplification factor. The Fermi–Dirac distribution function represents the Fermi–Dirac distribution, which can be expressed as [23]

(7) $$f_{d}\left(a\right)={\left(e^{\left(a-\mu_{c}\right)/k_{B}T}+1\right)}^{-1}$$

The inter-band conductivity *σ*_inter_ represents the leap process of the electron.

(8) $$\sigma_{\mathrm{inter}}=-\frac{ie^{2}\left(w-i2\Gamma \right)}{\pi \hbar^{2}}\int_{0}^{\infty }\frac{f_{d}\left(-a\right)-f_{d}\left(a\right)}{{\left(w-i2\Gamma \right)}^{2}-4{\left(a/\hbar \right)}^{2}}da=-\frac{ie^{2}\left(w-i2\Gamma \right)}{\pi \hbar^{2}}F_{2}$$

where *F*_2_ represents the simplification factor. Considering the electricity of graphene *σ*_g_ is a complex number, it also can be expressed as

(9) $$\sigma_{g}=\sigma_{\mathrm{gr}}+i\sigma_{\mathrm{gi}}$$

where *σ*_gr_ is the real part of the surface conductivity and *σ*_gi_ is the imaginary part of the surface conductivity.

#### 3.1.2. Dielectric Constant of Graphene

The dielectric constant of graphene can be derived from Maxwell’s equation, which is known to be

(10) $$\nabla \times H=\vec{J}+\frac{\partial \vec{D}}{\partial t}$$

By rewriting it in time-harmonic form, the above equation can be converted into

(11) $$\nabla \times H=\vec{J}+\frac{\partial \vec{D}}{\partial t}=\vec{J}-i\omega \varepsilon_{0}\vec{E}=\sigma_{3D}\vec{E}-i\omega \varepsilon_{0}\vec{E}=-i\omega \left(\varepsilon_{0}-\frac{\sigma_{3D}}{i\omega }\right)E=-i\omega \varepsilon E$$

where *σ*_3D_ is the three-dimensional conductivity of graphene. We can obtain *σ*_3D_ by dividing the two-dimensional conductivity of graphene by the thickness of graphene.

(12) $$\sigma_{3D}=\sigma_{2D}/t$$

where *t* represents the thickness of graphene. The two-dimensional conductivity of graphene can be written as *σ*_2D_ = *σ*_g_.

We can get the total dielectric constant.

(13) $$\varepsilon =\varepsilon_{0}-\frac{\sigma_{\mathrm{gi}}}{\omega t}+i\frac{\sigma_{\mathrm{gr}}}{\omega t}$$

The relative dielectric constant of graphene *ε*_g_ can be obtained as

(14) $$\varepsilon_{g}=\left(\varepsilon_{0}-\frac{\sigma_{2D}}{i\omega t}\right)/\varepsilon_{0}=1-\frac{\sigma_{2D}}{i\omega t\varepsilon_{0}}=1+\frac{\sigma_{2D}}{\omega t\varepsilon_{0}}=1-\frac{\sigma_{i}}{\omega t\varepsilon_{0}}+i\frac{\sigma_{r}}{\omega t\varepsilon_{0}}$$

The relative dielectric constant of *N*_g_-layer graphene is

(15) $$\varepsilon_{g}=\left(\varepsilon_{0}-\frac{\sigma_{2D}}{i\omega N_{g}t}\right)/\varepsilon_{0}=1+\frac{i\sigma_{2D}}{\omega tN_{g}\varepsilon_{0}}$$

Since graphene is very thin, when the thickness tends to 0, the real part of the dielectric constant of graphene *ε*_gr_ can be approximated as

(16) $$\varepsilon_{\mathrm{gr}}\approx -\frac{\sigma_{i}}{\omega t\varepsilon_{0}}$$

The imaginary part of the dielectric constant *ε*_gi_ can be expressed as

(17) $$\varepsilon_{\mathrm{gi}}=\frac{\sigma_{r}}{\omega t\varepsilon_{0}}$$

The real part of the dielectric constant is determined by the imaginary part of the conductivity, and the imaginary part of the dielectric constant is determined by the real part of the conductivity.

By substituting the relationship between conductivity and dielectric constant, we can obtain the relationship between the dielectric constant of graphene and the frequency

(18) $$\varepsilon_{g}\left(\omega \right)=\varepsilon_{0}-\frac{e^{2}}{\pi \hbar^{2}\left(w-i2\Gamma \right)\omega t}F_{1}+\frac{e^{2}\left(w-i2\Gamma \right)}{\pi \hbar^{2}\omega t}F_{2}$$

#### 3.1.3. Magnetic Permeability and Refractive Index of Graphene

The propagation speed of electromagnetic waves in the medium can be expressed as

(19) $$\begin{array}{l} v=c/\sqrt{\varepsilon_{r}\mu_{r}}\\ v=c/n \end{array}$$

where *c* is the velocity of the electromagnetic wave in a vacuum. According to the relationship between *c* and *v*, the refractive index *n* can be calculated as

(20) $$n=\sqrt{\varepsilon_{r}\mu_{r}}$$

It is known that the magnetic permeability of graphene *μ*_g_ can be taken as 1 [24]. The complex refractive index of graphene can be simplified as

(21) $$n_{g}\left(\omega \right)=\sqrt{\varepsilon_{0}-\frac{e^{2}}{\pi \hbar^{2}\omega t}\left[\frac{F_{1}}{\left(w-i2\Gamma \right)}+\left(w-i2\Gamma \right)F_{2}\right]}$$

Since the refractive index of graphene is a complex number, the relationship between the refractive index and the dielectric constant can be expressed as

(22) $$\begin{array}{l} n_{\mathrm{gr}}^{2}-n_{\mathrm{gi}}^{2}=\varepsilon_{\mathrm{gr}}\\ 2n_{\mathrm{gr}}n_{\mathrm{gi}}=\varepsilon_{\mathrm{gi}} \end{array}$$

By analyzing the relationship between graphene conductivity and complex refractive index, the complex refractive index can be expressed by electrical conductivity.

(23) $$n_{g}^{2}\varepsilon_{0}\omega t=-\sigma_{\mathrm{gi}}+i\sigma_{\mathrm{gr}}$$

The above equations mean that the square of the refractive index is the relative dielectric constant.

### 3.2. Electromagnetic Properties of Copper

Since copper is a conductor, the relative dielectric constant of copper ε_cu_ is 1, the relative permeability μ_cu_ of copper is 1, and the conductivity of copper α_cu_ is 5.998 × 10^7^ S/m. The above parameters are generally specific to a particular standard. For example, when the conductivity of copper is measured, the temperature is the standard temperature (25 °C).

## 4. Analysis of Influencing Factors

This section analyzes the influencing factors of the electromagnetic properties of graphene and copper. This analysis is mainly focused on the electromagnetic properties of graphene. Considering influencing factors such as scattering rate, electrochemical potential, temperature, and frequency, a theoretical qualitative analysis was performed. The quantitative analysis of each influencing factor will be carried out in the next calculation chapter.

### 4.1. Scattering Rate

The scattering rate of graphene Γ is related to the relaxation time τ, and the relationship can be expressed as Γ = 1/2*τ*. The relaxation time is used to represent scattering rate in the subsequent research. The relaxation time τ of graphene is related to its carrier mobility *n*_c_, and the relationship is *τ* = *n*_c_*μ*_c_/(*ev*_F_^2^) [24]. *v*_F_ is the Fermi velocity. In this paper, the value of the Fermi rate is 10^6^ m/s and the carrier mobility of graphene is 2 m^2^/V·s.

### 4.2. Electrochemical Potential

In the ideal case, the chemical potential *µ*_c_ of thin graphene layer can be determined by the carrier concentration *n*_s_ [24]:

(24) $$n_{s}=2/\pi \hbar^{2}v_{F}^{2}\int_{0}^{\infty }a\left[f_{d}\left(a\right)-f_{d}\left(a+2\mu_{c}\right)\right]da$$

The carrier concentration can be adjusted by chemical doping or gate bias.

### 4.3. Temperature

Temperature affects the motion of electrons, which will change the conductivity of graphene. However, we generally consider only room temperature T = 300 K.

### 4.4. Frequency

The same electromagnetic wave has different wavelengths in different mediums, which in turn affects the graphene conductivity. Frequency can be expressed as *w* = 2π*f*. For better analysis of electromagnetic properties, we consider a wide range of frequency bands of the electromagnetic wave. Therefore, we chose to analyze the full frequency band from Hz to THz. The selected frequency bands are shown in Table 1 below.

### 4.5. Factors Influencing the Electromagnetic Properties of Copper

Since copper is a conductor, for its electromagnetic properties, we mainly analyzed the conductivity. The conductivity of copper can be generally affected by the temperature, the degree of doping, and the processing method. As for temperature, the conductivity of copper decreases as the temperature increases. As for the degree of doping, the impurities in copper have a great influence on the conductivity of copper. As for the processing method, it mainly includes cold processing and heat treatment.

## 5. Computational Analysis

In this section, the conductivity, dielectric constant, and refractive index of graphene are calculated by MATLAB under different influencing factors. Additionally, we obtained the curves of the electromagnetic properties with frequency when the temperature was taken as 100 K, 200 K, 300 K, 400 K, and 500 K; the electrochemical potential taken as 0.1 eV, 0.2 eV, 0.3 eV, 0.4 eV, and 0.5 eV; and the relaxation time taken as 1 ps, 2 ps, 3 ps, 4 ps, and 5 ps, respectively. These analyses are based on the full frequency band, ranging from Hz to THz.

### 5.1. Electrical Conductivity

Fixing temperature as 300 K, relaxation time as 3 ps, we analyze the variation of real and imaginary parts of graphene conductivity with frequency, when the electrochemical potential is taken as 0.1 eV, 0.2 eV, 0.3 eV, 0.4 eV, and 0.5 eV, respectively.

**Figure 2:**
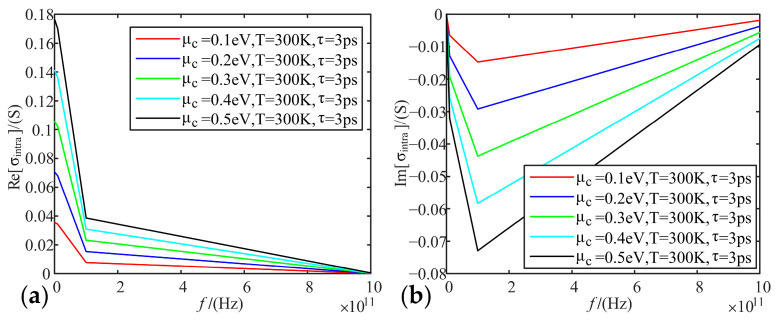
Variation of graphene intra-band conductivity with frequency at different electrochemical potentials: (**a**) real part; (**b**) imaginary part.

**Figure 3:**
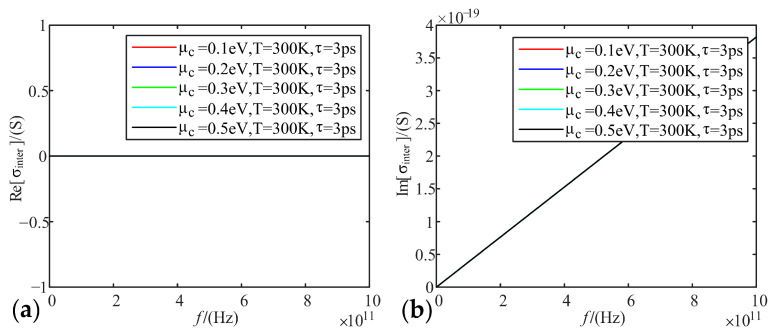
Variation of graphene inter-band conductivity with frequency at different electrochemical potentials: (**a**) real part; (**b**) imaginary part.

**Figure 4:**
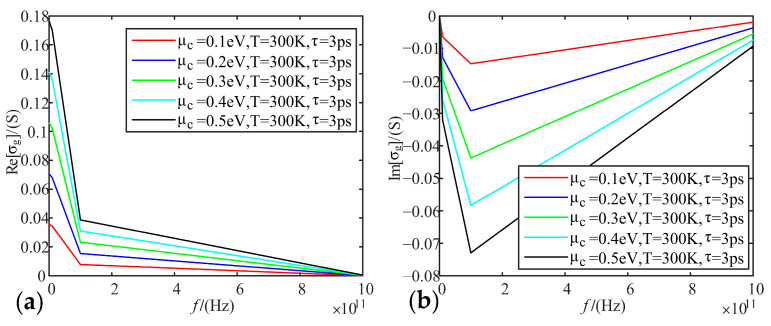
Variation of graphene total conductivity with frequency at different electrochemical potentials: (**a**) real part; (**b**) imaginary part.

Figure 2, Figure 3 and Figure 4 show that the intra-band conductivity contributes more to the conductivity and the inter-band conductivity can be neglected. Therefore, we chose to neglect the inter-band conductivity in the later study. The real part of the conductivity decreases with increasing frequency and begins to decrease slowly after 100 GHz. However, the imaginary part decreases firstly and then increases at 100 GHz. The real and imaginary parts both change abruptly after the frequency increases to 100 GHz. On the other hand, in terms of the effect of electrochemical potential on the conductivity, the higher the electrochemical potential, the larger the real part of the conductivity and the smaller the imaginary part.

Fixing electrochemical potential is 0.3 eV, and relaxation time as 3 ps; we calculated the variation of the real and imaginary parts of graphene conductivity with frequency, when the temperature was taken as 100 K, 200 K, 300 K, 400 K, and 500 K, respectively.

**Figure 5:**
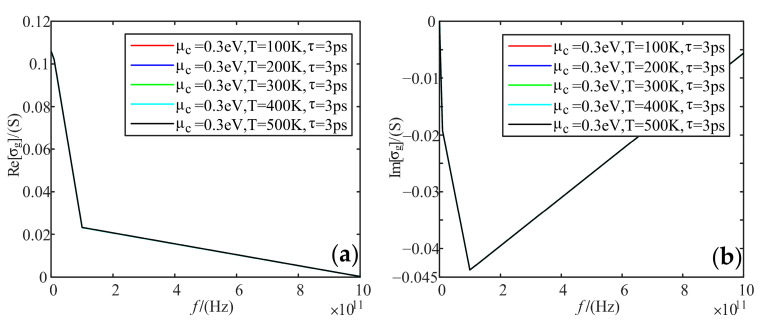
Variation curve of graphene total conductivity with frequency at different temperatures: (**a**) real part; (**b**) imaginary part.

Figure 5 shows that temperature change does not affect the real and imaginary part of graphene conductivity.

Fixing electrochemical potential is 0.3 eV, and the temperature is 300 K; we analyzed the variation of real and imaginary parts of graphene conductivity with frequency, when the relaxation time was taken as 1 ps, 2 ps, 3 ps, 4 ps, and 5 ps, respectively.

**Figure 6:**
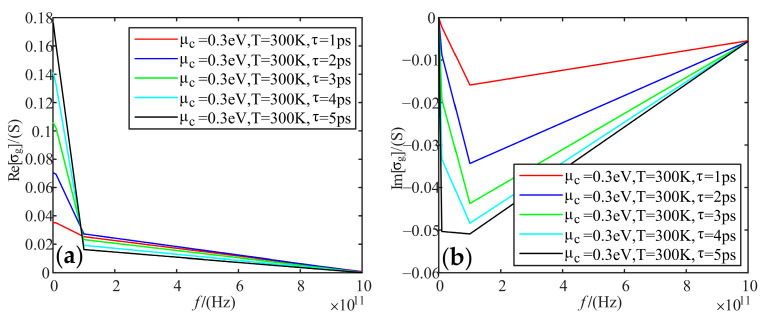
Variation of graphene total conductivity with frequency at different relaxation times: (**a**) real part; (**b**) imaginary part.

Figure 6 shows that the real part of conductivity decreases with increasing frequency. In particular, the slope of the real part changes more abruptly at 100 GHz. Meanwhile, when the frequency is less than 100 GHz, the real part of conductivity increases with the increasing relaxation time. When the frequency is greater than 100 GHz, the real part of conductivity is the largest when the relaxation time is 2 ps, followed by 1 ps, and then 3 ps, 4 ps, and 5 ps. As for the imaginary part of the conductivity, it decreases with increasing relaxation time. Unlike the real part, when the frequency is above 100 GHz, the imaginary part increases with the increasing frequency.

### 5.2. Dielectric Constant

Fixing temperature is 300 K, and the relaxation time is 3 ps; we analyzed the variation of the real and imaginary parts of the dielectric constant of graphene with frequency when the electrochemical potential was taken as 0.1 eV, 0.2 eV, 0.3 eV, 0.4 eV, and 0.5 eV, respectively.

**Figure 7:**
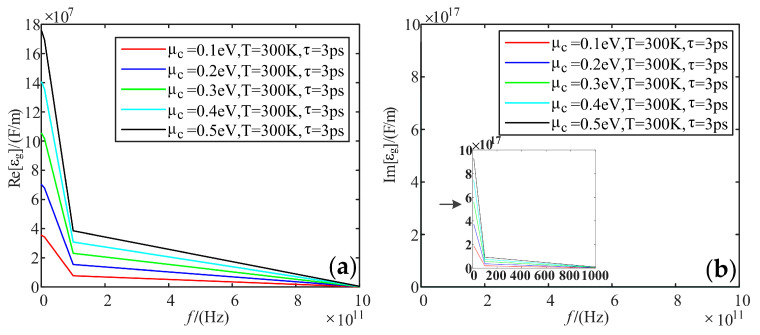
Variation curve of graphene dielectric constant with frequency at different electrochemical potentials: (**a**) real part; (**b**) imaginary part.

Figure 7 shows that the real part decreases with the increasing frequency and slows down at 100 GHz. The overall increases with the increasing electrochemical potential. As for the imaginary part, it has a huge value in the low-frequency band, up to 9.3 * 10^17^ F/m, and its value tends to be 0 in the high-frequency band. The effect of electrochemical potential is the same as the real part.

Fixing electrochemical potential is 0.3 eV, and the relaxation time is 3 ps; we analyzed the variation of the real and imaginary parts of the dielectric constant of graphene with frequency when the temperature was taken as 100 K, 200 K, 300 K, 400 K, and 500 K, respectively.

**Figure 8:**
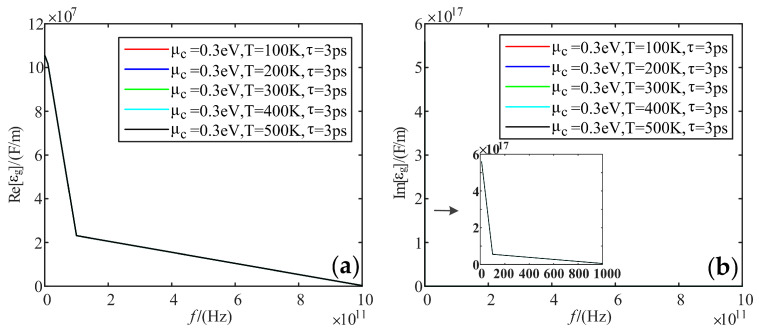
Variation curve of graphene dielectric constant with frequency at different temperatures: (**a**) real part; (**b**) imaginary part.

Figure 8 shows that the real and imaginary parts of the dielectric constant are not affected by temperature.

Fixing electrochemical potential is 0.3 eV, and the temperature is 300 K; we analyzed the variation of the real and imaginary parts of the dielectric constant of graphene with frequency, when the relaxation time was taken as 1 ps, 2 ps, 3 ps, 4 ps, and 5 ps.

**Figure 9:**
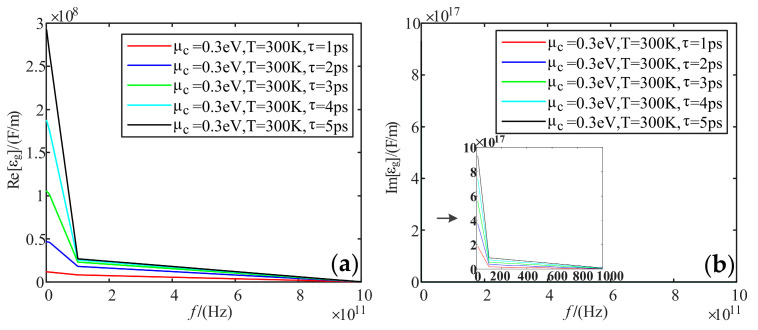
Variation curve of graphene dielectric constant with frequency at different relaxation times: (**a**) real part; (**b**) imaginary part.

Figure 9 shows that the real part of the dielectric constant increases with the increasing relaxation time. Meanwhile, when the frequency reaches 100 GHz, the real part of the dielectric constant begins to decrease slowly. The imaginary part of the dielectric constant reaches up to 9.4 × 10^17^ F/m at lower frequencies and tends to 0 at higher frequency bands. The effect of the relaxation time is the same as the real part.

### 5.3. Refractive Index

Fixing temperature is 300 K, and the relaxation time is 3 ps; we analyzed the variation of the real and imaginary parts of the refractive index of graphene with frequency when the electrochemical potential was taken as 0.1 eV, 0.2 eV, 0.3 eV, 0.4 eV, and 0.5 eV, respectively.

**Figure 10:**
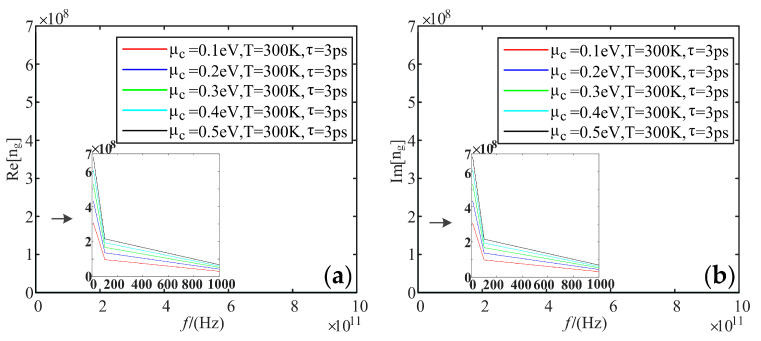
Variation curve of the refractive index of graphene with frequency at different electrochemical potentials: (**a**) real part; (**b**) imaginary part.

Figure 10 shows that the real and imaginary parts of the refractive index are up to 6.8 × 10^8^ F/m at low frequencies and tend to be 0 at high frequencies. Additionally, these two parts increase with the increasing electrochemical potential.

Fixing electrochemical potential is 0.3 eV, and the relaxation time is 3 ps; we analyzed the variation of the real and imaginary parts of the refractive index of graphene with frequency when the temperature was taken as 100 K, 200 K, 300 K, 400 K, and 500 K, respectively.

**Figure 11:**
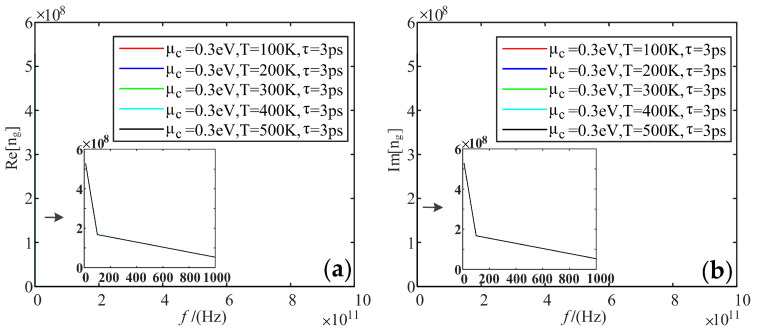
Variation curve of the refractive index of graphene with frequency at different temperatures: (**a**) real part; (**b**) imaginary part.

Figure 11 shows that the real and imaginary parts of the refractive index reach up to 5.2 × 10^8^ F/m at low frequencies, and both tend to be 0 at higher frequency bands. The effect of temperature is not reflected.

Fixing electrochemical potential is 0.3 eV, and the temperature is 300 K; we analyzed the variation of the real and imaginary parts of the refractive index of graphene with frequency, when the relaxation time was taken as values of 1 ps, 2 ps, 3 ps, 4 ps, and 5 ps, respectively.

**Figure 12:**
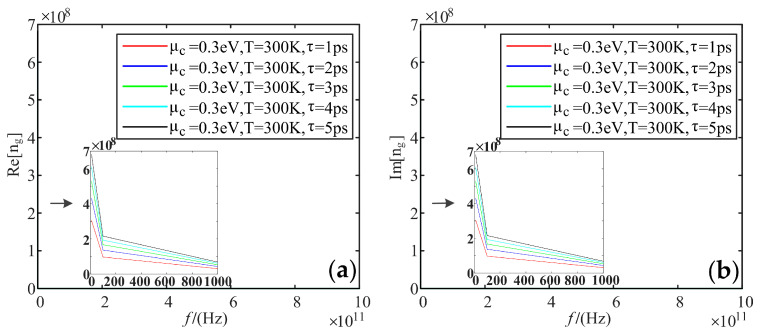
Variation curve of the refractive index of graphene with frequency at different relaxation times: (**a**) real part; (**b**) imaginary part.

Figure 12 shows that the real and imaginary parts of the refractive index reach up to 6.8 × 10^8^ F/m at low frequencies, and both tend to be 0 at high-frequency bands. These two parts increase with the effect of relaxation time.

According to Figure 4, Figure 5, Figure 6, Figure 7, Figure 8, Figure 9, Figure 10, Figure 11 and Figure 12, we can conclude the relationship between electromagnetic properties and influencing factors. This result can lay the foundation for further tuning the electromagnetic properties of graphene. The relationship is shown in Table 2.

Table 2 shows the relationship between electromagnetic properties and influencing factors, where ‘\’ represents no relation; ‘+’ represents proportional relation; ‘−’ represents inversely proportional relation; and ‘− +’ represents firstly proportional relation and then inversely proportional relation.

## 6. Prediction Models of Electromagnetic Properties

By analyzing the variation relationship of electromagnetic properties of the above electrode plate materials, we can see the existence of multiple iterations and relatively independent relationships between different influencing factors. When solving electromagnetic properties of graphene PD sensor, to further simplify electromagnetic properties calculations and facilitate engineering applications, this section establishes multiple linear regression models of three electromagnetic properties based on the least-square method.

### 6.1. Prediction Model of Conductivity

**Figure 13:**
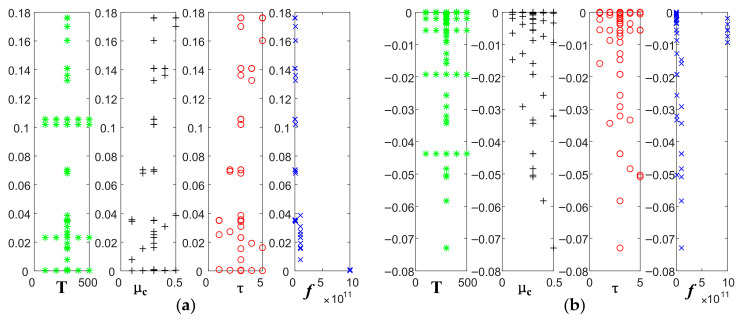
Linear correlation between T, μ_c_, τ, *f*, and conductivity: (**a**) real part; (**b**) imaginary part.

Based on the conductivity, a dataset with four different influencing factors as independent variables and conductivity as the dependent variable was constructed. Additionally, a scatter plot was created to view the linearity correlation between the independent variables and the predictor variables. The linearity is shown in Figure 13.

Next, the four independent variables of temperature, electrochemical potential, relaxation time, and frequency were used to establish multiple linear regression equations with conductivity, and the regression coefficients corresponding to the four independent variables were calculated using the least-squares method. The real and imaginary regression equations of conductivity are as follows.

(25) $$\begin{array}{l} \sigma_{gr}\left(T,\mu_{c},\tau ,f\right)=-0.08+4.04T+0.30\mu_{c}+0.03\tau -1.06\cdot 10^{-13}f\\ \sigma_{gi}\left(T,\mu_{c},\tau ,f\right)=0.01-3.50\cdot 10^{-9}T-0.02\mu_{c}-0.002\tau -3.8\cdot 10^{-15}f \end{array}$$

### 6.2. Prediction Model of Dielectric Constant

**Figure 14:**
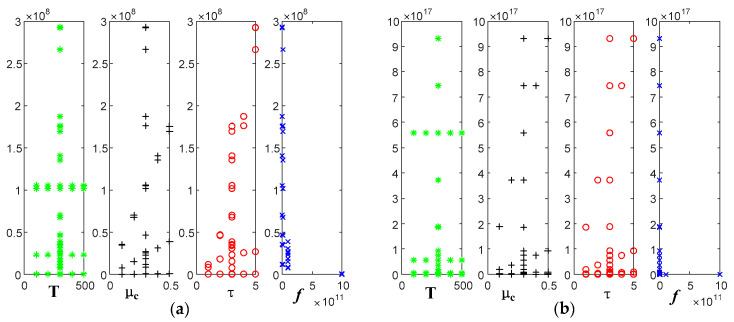
Linear correlation between T, μ_c_, τ, *f*, and dielectric constant: (**a**) real part; (**b**) imaginary part.

Based on the dielectric constant, a dataset with four different influencing factors as independent variables and dielectric constant as the dependent variable was constructed. Additionally, a scatter plot was created to view the linearity correlation between the independent variables and the predictor variables. The linearity is shown in Figure 14.

Next, the four independent variables of temperature, electrochemical potential, relaxation time, frequency, and dielectric constant are established as multiple linear regression equations. The regression coefficients corresponding to the four independent variables are calculated using the least-squares method. The regression equations for the real and imaginary parts of the dielectric constant are as follows:

(26) $$\begin{array}{l} \varepsilon_{gr}\left(T,\mu_{c},\tau ,f\right)=-1.58\cdot 10^{8}+58.77T+2.97\cdot 10^{8}\mu_{c}+5.83\cdot 10^{7}\tau -0.001f\\ \varepsilon_{gi}\left(T,\mu_{c},\tau ,f\right)=-4.57+2.96\cdot 10^{10}T+1.72\cdot 10^{17}\mu_{c}+1.72\cdot 10^{16}\tau -63362f \end{array}$$

### 6.3. Prediction Model of Refractive Index

**Figure 15:**
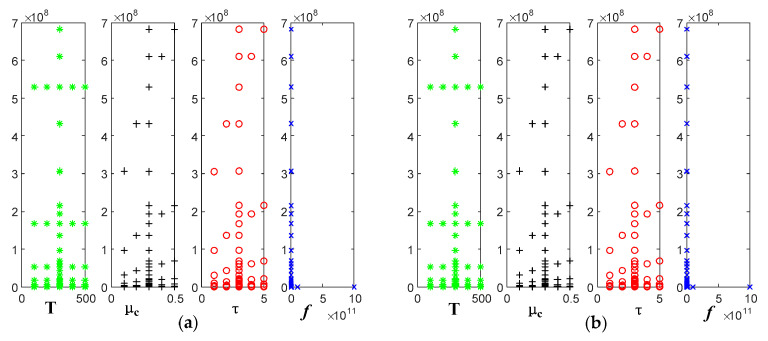
Linear correlation between T, μ_c_, τ, *f* and refractive index (**a**) Real part; (**b**) Imaginary part.

Based on the refractive index, a dataset with four different influencing factors as independent variables and a refractive index as the dependent variable was constructed. Additionally, a scatter plot was created to view the linearity correlation between the independent variables and the predictor variables. The linearity is shown in Figure 15.

Next, the four independent variables of temperature, electrochemical potential, relaxation time, and frequency were used to establish the multiple linear regression equation with the refractive index. The regression coefficients corresponding to the four independent variables were calculated using the least-squares method. The regression equations of refractive index real part imaginary part are as follows.

(27) $$\begin{array}{l} n_{gr}\left(T,\mu_{c},\tau ,f\right)=2.08\cdot 10^{6}+19.47T+1.13\cdot 10^{8}\mu_{c}+1.14\cdot 10^{7}\tau -7.72\cdot 10^{-5}f\\ n_{gi}\left(T,\mu_{c},\tau ,f\right)=2.08\cdot 10^{6}+19.43T+1.13\cdot 10^{8}\mu_{c}+1.14\cdot 10^{7}\tau -7.72\cdot 10^{-5}f \end{array}$$

## 7. Simulation and Experimental Analysis

### 7.1. Simulation Analysis

**Figure 16:**
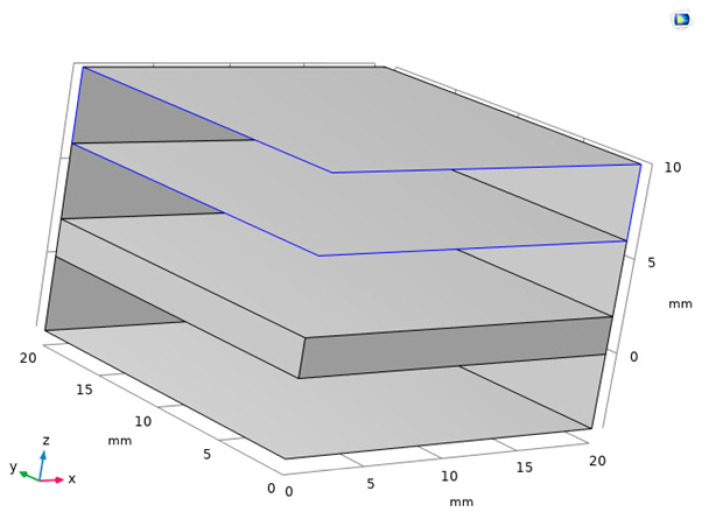
Traditional copper electrode plate model.

**Figure 17:**
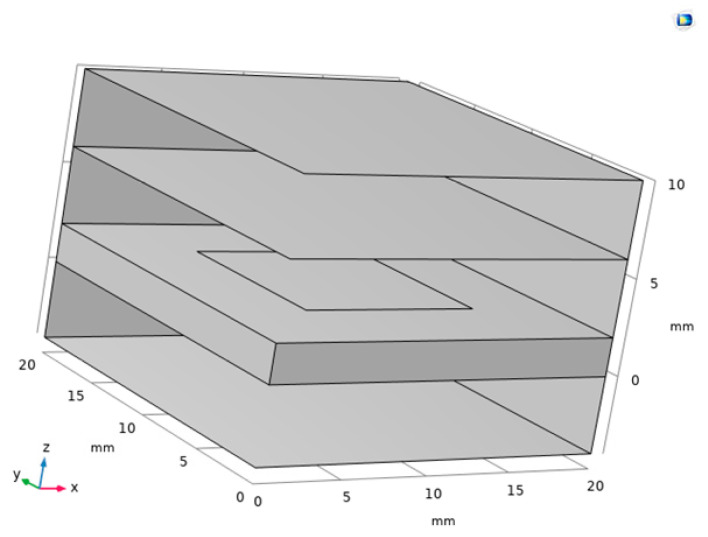
Graphene copper-clad electrode plate model.

The simulation analysis uses the electromagnetic properties we derived, to construct finite element models of the traditional copper electrode plate and graphene copper-clad electrode plate, respectively. The simulation parameters are shown in Table 3:

Figure 16 is the traditional copper electrode plate model. Figure 17 is the graphene copper-clad electrode plate model. These two models consist of a perfect matching layer, air layer, electrode plate, and air layer.

In Figure 17, the middle area of the electrode plate has a layer of graphene on the surface.

**Figure 18:**
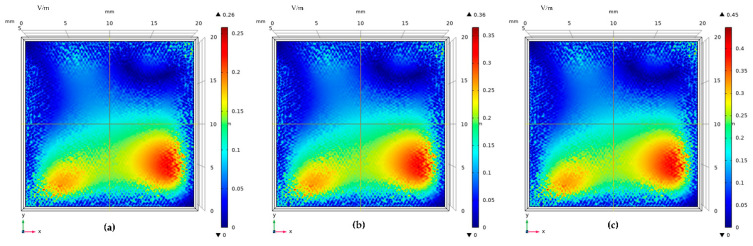
Surface electric field distribution of traditional copper electrode plate: (**a**) f = 30 MHz; (**b**) f = 60 MHz; and (**c**) f = 90 MHz.

**Figure 19:**
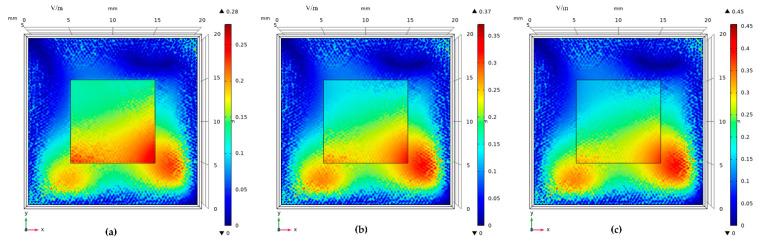
Surface electric field distribution of graphene copper-clad electrode plate: (**a**) f = 30 MHz; (**b**) f = 60 MHz; and (**c**) f = 90 MHz.

When partial discharge signals of 30 MHz, 60 MHz, and 90 MHz are incident, respectively, the response of different models to the signal is different. Figure 18 is the surface electric field distribution of the traditional electrode plate model. Figure 19 is the surface electric field distribution of the graphene copper-clad electrode plate model.

It can be seen that the electric field distribution on the surface of the graphene copper-clad electrode plate is significantly higher than that of the traditional copper electrode plate. This is because of the better absorption of the electromagnetic wave signal generated by partial discharge. This simulation can verify that the graphene partial discharge sensor can couple more electromagnetic than the traditional partial discharge sensor.

### 7.2. Experimental Analysis

**Figure 20:**
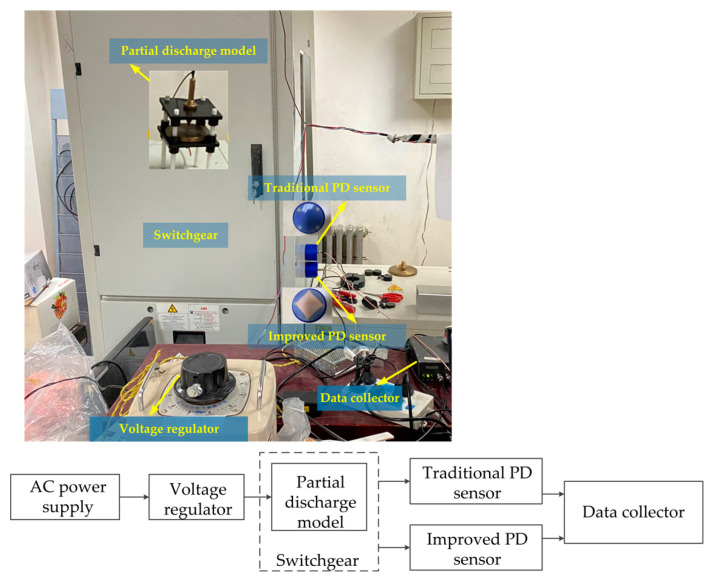
Experimental setup and schematic diagram.

**Figure 21:**
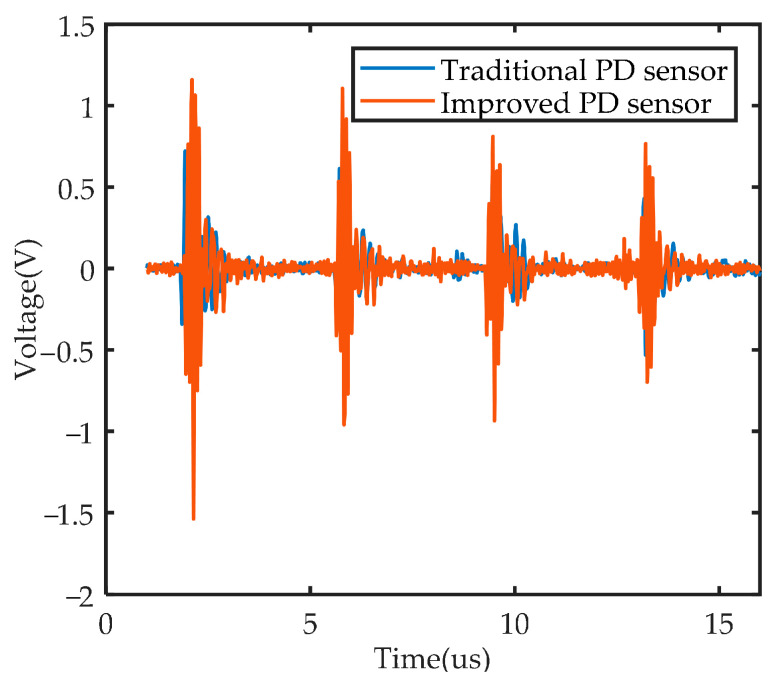
Experimental result of PD detection.

A partial discharge detection system was established in the laboratory, which includes AC power supply, voltage regulator, switchgear with partial discharge defect model placed, traditional partial discharge sensor, improved partial discharge sensor, and data acquisition device. The electrode plate of the traditional partial discharge sensor is copper, and the electrode plate of the improved sensor is graphene copper-clad electrode plate instead, which is processed by the commissioned manufacturer. The experimental setup and schematic diagram are shown in Figure 20. During the experiment, the voltage regulator was adjusted to 55 kV, and the partial discharge model can be heard to start discharging. The partial discharge pulses detected by the two sensors are collected and compared. The four discharge pulse waveforms within 15 us are shown in Figure 21.

Figure 21 shows that for the same discharge pulse, the improved partial discharge sensor has a higher gain than the traditional partial discharge sensor. It can be seen that the introduction of graphene can indeed improve the gain of the partial discharge sensor. The experimental result is consistent with the simulation result that the signal coupled to the surface of the sensor electrode plate is enhanced.

## 8. Conclusions

Aiming at the improvement of traditional PD sensor materials, this paper proposes a method to analyze the electromagnetic properties of the electrode plate material of the graphene PD sensor. This method can lay the foundation for further analysis of the electromagnetic wave propagation law and further design of PD sensor material structure. The following conclusions are obtained:

(1) As for the conductivity, the higher the frequency, the smaller the real part of the conductivity, and the imaginary part firstly decreases and then increases. The higher the electrochemical potentials, the larger the real part of the conductivity, and the smaller the imaginary part. Temperature does not affect the real and imaginary part of the conductivity of graphene. When the relaxation time increases, the real part of the conductivity increases, and the imaginary part decreases;
(2) As for the dielectric constant, the higher the frequency, the less the real part gradually decreases. The imaginary part has a huge value in the low-frequency band, and its value tends to 0 in the high-frequency band. The higher the electrochemical potentials, the larger the real part of the dielectric constant. Temperature does not affect the real and imaginary parts of the dielectric constant. When the relaxation time increases, the real part and imaginary part of the dielectric constant increase;
(3) As for the refractive index, because of the large frequency span of the sampling frequency band, the real and imaginary parts of the refractive index are larger when the frequency is small, but the high-frequency band tends to be 0. Additionally, the real and imaginary parts increase with the electrochemical potentials and relaxation time, and these two parts cannot reflect the effects of temperature.
(4) A multiple linear regression model based on the least-squares method is established for the three electromagnetic properties, which can further simplify the calculation of electromagnetic properties and provide a good fit to the data.
(5) The simulation and experiment verify that the graphene partial discharge sensor can better absorb the partial discharge signal compared with the traditional partial discharge sensor.

This article mainly focuses on the electromagnetic properties of the electrode plate material of the graphene partial discharge sensor and verifies the feasibility of graphene partial discharge sensors. In future work, it will be important to know how to modulate the electromagnetic properties of graphene around the four factors and find the optimal parameters for the optimization and customization of the sensor. Additionally, we will further deepen the design of the graphene partial discharge sensor from the micromechanical aspects for the graphene-copper composite using the first principle and experiments.

## Figures and Tables

**Table 1:** Full frequency band sampling.

Hz	kHz	MHz	GHz	THz
1 Hz, 10 Hz	1 kHz, 10 kHz, 100 kHz	1 MHz, 10 MHz, 100 MHz	1 GHz, 10 GHz, 100 GHz	1 THz

**Table 2:** The relationship between electromagnetic properties and influencing factors.

Electromagnetic Properties	Part	T	μ_c_	τ	*f*
Conductivity	Re	\	+	+	−
	Im	\	−	−	− +
Dielectric constant	Re	\	+	+	−
	Im	\	+	+	−
Refractive index	Re	\	+	+	−
	Im	\	+	+	−

**Table 3:** Simulation parameters.

Material	T (K)	μ_c_ (eV)	τ (fs)	*F* (MHz)	σ (S/m)	ε
Graphene	300	0.5	65	30, 60, 90	\	\
Copper	\	\	\	30, 60, 90	5.998 × 10^7^	1

## Data Availability

Not applicable.

## Nomenclature

σ,ε,μ,n	Electrical conductivity, dielectric constant, magnetic permeability, and refractive index
w,f	Frequency, *w* = 2π*f*
$\mu_{c}$	Electrochemical potential
Γ	Scattering rate
τ	Relaxation time
T	Temperature
i	Imaginary unit
e	Electron charge
h	Planck constant
ħ	Angular Planck constant, *ħ* = *h*/2π
a	Energy
$f_{d}\left(a\right)$	Fermi–Dirac distribution function
$F_{1}$	Simplification factor. $F_{1}=\int_{0}^{\infty }a\left(\frac{\partial f_{d}\left(a\right)}{\partial a}-\frac{\partial f_{d}\left(-a\right)}{\partial a}\right)da$
$F_{2}$	Simplification factor. $F_{2}=\int_{0}^{\infty }\frac{f_{d}\left(-a\right)-f_{d}\left(a\right)}{{\left(w-i2\Gamma \right)}^{2}-4{\left(a/\hbar \right)}^{2}}da$
$\varepsilon_{0}$	Vacuum dielectric constant. ε_0_ = 8.854187817 × 10^−12^ F/m
$\mu_{0}$	Vacuum magnetic permeability. *μ*_0_ = 4π × 10^−7^ H/m
$\varepsilon_{r}$	Relative dielectric constant
$\mu_{r}$	Relative magnetic permeability
t	Thickness
$\sigma_{3D}$	Three-dimensional conductivity
$\sigma_{2D}$	Two-dimensional conductivity. *σ*_2*D*_ = *σ*_3*D*_/*t*
$\sigma_{g}$	Electricity of graphene. *σ*_2*D*_ = *σ*_*g*_
$\sigma_{gr}$	The real part of the graphene conductivity
$\sigma_{gi}$	The imaginary part of the graphene conductivity
$\varepsilon_{g}$	The dielectric constant of graphene
$\varepsilon_{gr}$	The real part of the graphene dielectric constant
$\varepsilon_{gi}$	The imaginary part of the graphene dielectric constant
$\mu_{g}$	The magnetic permeability of graphene
$n_{g}$	Refractive index of graphene
$n_{\mathrm{gr}}$	The real part of the graphene refractive index
$n_{\mathrm{gi}}$	The imaginary part of the graphene refractive index
$\sigma_{\mathrm{intra}}$	Intra-band conductivity
$\sigma_{\mathrm{inter}}$	Inter-band conductivity
$N_{g}$	Graphene layers number
$k_{B}$	Boltzmann constant
v	The propagation speed of electromagnetic waves in the medium
$n_{s}$	Carrier concentration
c	The velocity of the electromagnetic wave in a vacuum
$v_{F}$	Fermi velocity
